# Visual rehabilitation via microperimetry in patients with geographic atrophy: a pilot study

**DOI:** 10.1186/s40942-017-0071-1

**Published:** 2017-05-22

**Authors:** Juan Abel Ramírez Estudillo, Mario Isaías León Higuera, Sergio Rojas Juárez, Maria de Lourdes Ordaz Vera, Yessica Pablo Santana, Benito Celis Suazo

**Affiliations:** 1 Retina Department, Fundación Hospital Nuestra Señora de la Luz, Ezequiel Montes 135, Cuauhtemoc, Tabacalera, 06030 Ciudad de México, México; 2 Retina and Vitreous Research Fellow, Fundación Hospital Nuestra Señora de la Luz, Ezequiel Montes 135, Cuauhtemoc, Tabacalera, 06030 Ciudad de México, México; 3 Low Vision Department, Fundación Hospital Nuestra Señora de la Luz, Ezequiel Montes 135, Cuauhtemoc, Tabacalera, 06030 Ciudad de México, México

**Keywords:** Age-related macular degeneration, Geographic atrophy, Visual rehabilitation, Microperimetry, Fixation stability

## Abstract

**Background:**

Age-related macular degeneration (AMD) is the leading cause of blindness in the western world. As a consequence of AMD, patients develop structural damage that comprises the fovea and subsequently present loss of central vision, low visual acuity and unstable fixation. Contrary to what happens with anti-angiogenic treatment in neovascular AMD, there is currently no definitive treatment to reverse geographic atrophy progression. The aim of this study was to determine the effectiveness of the visual rehabilitation treatment via microperimetry in patients with geographic atrophy.

**Methods:**

Longitudinal and prospective study, 18 patients with areas of geographic atrophy in their eye of better visual acuity were included. Macular integrity assessment (Maia) microperimeter (CentreVue, Padova, Italy) was used to diagnose retinal fixation and sensitivity in these patients. Based on these data and using the training module available in the equipment, the patients underwent visual rehabilitation sessions intended to allow the patient to establish the best possible fixation in the best area of retinal sensitivity. To determine the training effectiveness, the following variables were compared before and after: visual acuity in LogMAR scale with ETDRS charts, reading speed with Minnesota Low-Vision Reading Test (MN Read), average sensitivity threshold in microperimetry; P1 and 95% Bivariate Contour Ellipse Area (BCEA) values were used for fixation stability measurement.

**Results:**

Mean age was 77 years old (65–92). Visual acuity of the trained eye was on average 0.7 versus 0.6 LogMAR (*p* = 0.006) before and one week after training. Reading speed, using both eyes, was 47 words per minute (wpm) before training and 69 wpm after training (*p* = 0.04). Average retinal sensitivity was 14.1 versus 14.6 db (*p* = 0.4). Fixation stability improved with P1 values of 45% versus 51% (*p* = 0.05) and 95% BCEA values of 43 versus 25 (*p* = 0.02) before and after training, respectively.

**Conclusions:**

Visual training via microperimetry in patients with age-related macular degeneration is effective in improving fixation stability, reading speed, and visual acuity, measured one week after training is completed.

## Background

Age-related macular degeneration (AMD) is the leading cause of blindness in the western world [1]. As a consequence of AMD, patients develop structural damage that comprises the fovea and subsequently present loss of central vision, low visual acuity and unstable fixation. AMD is the leading cause of irreversible loss of central vision for people over 50 years of age in the United States [2].

Contrary to what happens with anti-angiogenic treatment in wet macular degeneration, there is currently no definitive treatment to reverse geographic atrophy progression [3]. Recent clinical trials investigated or are developing therapies through multiple modalities including complement system and inflammation, visual cycle modulation, neuroprotection and cell replacement therapy [4]. Therefore, till date, low vision optical aids and eccentric viewing training techniques are still the only options that can be offered as a treatment in most patients.

**Fig. 1:**
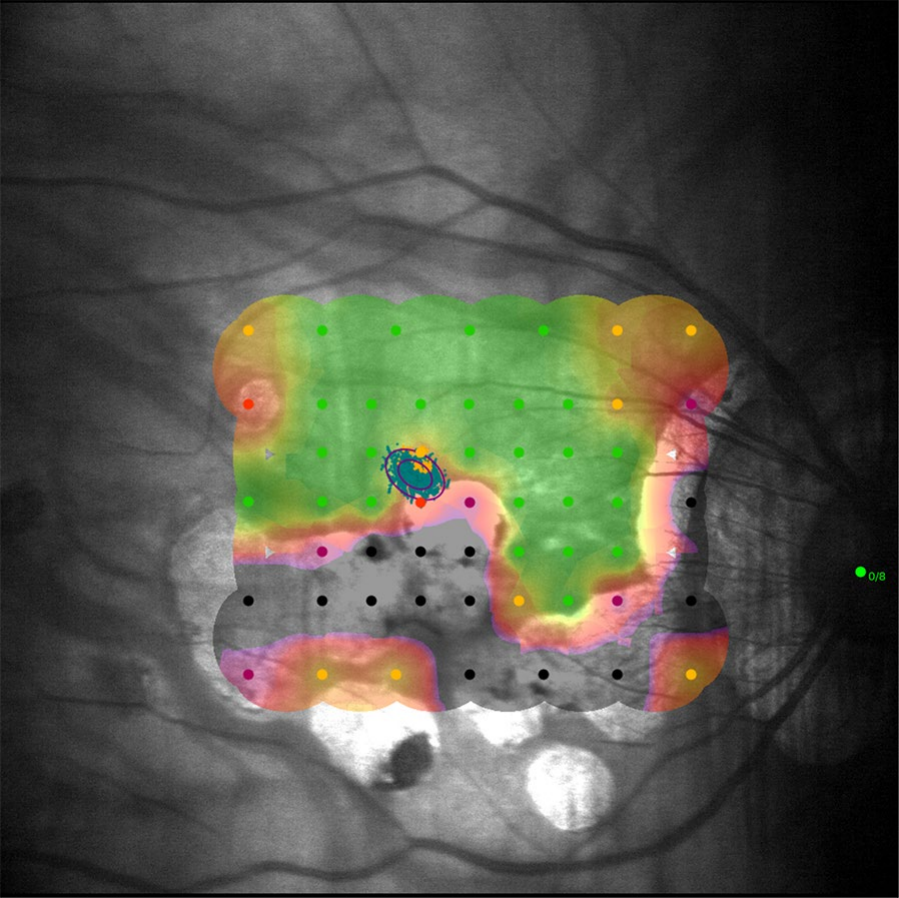
**Right eye microperimetry.** Stable extra-foveal fixation with good sensitivity area (*green zone*) in a patient with myopic maculopathy

Patients with eccentric fixation and low vision use extrafoveal areas of the retina to compensate loss of central fixation; these areas of the retina are known as Preferred Retinal Loci (PRL). Many patients use a PRL in healthy areas of peripheral macula; however, this location is not always ideal and fixing stability is not the best [5]. It has been described that fixation can be trained and it is possible to establish new fixation points in patients with loss of central vision (Fig. 1). Microperimetry systems with biofeedback training have been used for visual rehabilitation and for improving fixation stability in patients with eccentric vision [6].

The aim of this study was to determine if a rehabilitation process with training of eccentric fixation via microperimetry caused improvement of visual function in patients with geographic atrophy related to AMD.

## Methods

### Materials and methods

Patients with areas of geographic atrophy in their eye of better visual acuity were recruited without discriminating against age or sex. Patients voluntarily accepted to participate in the study and signed the informed consent. Ethics Committee approval was obtained and research adhered to the tenets of the Declaration of Helsinki.

Patients with cataract, other concomitant macular diseases such as diabetic retinopathy, and other causes of visual loss different to macular atrophic changes in both eyes were excluded from the study.

**Fig. 2:**
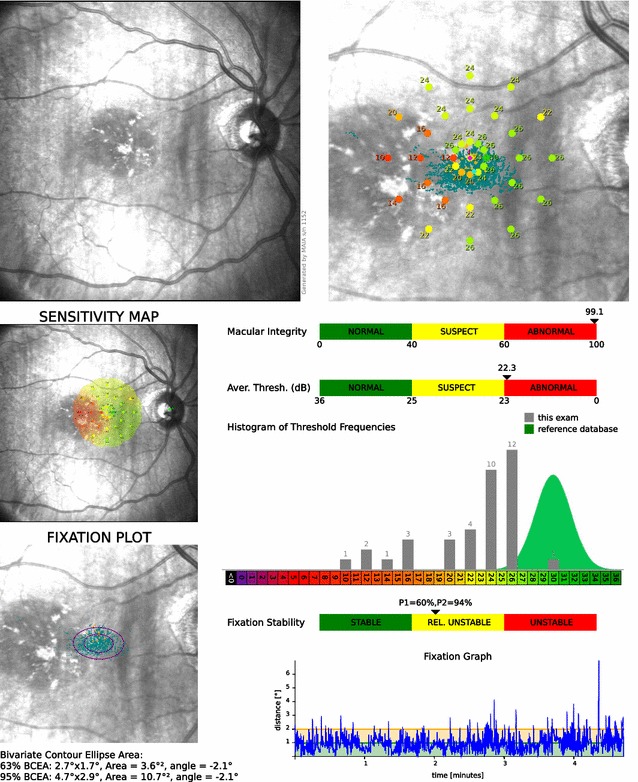
**Microperimetry report**. Sensitivity map and fixation areas as well as normative scales expressed in *colors*. Look at extra-foveal fixation used by the patient

All patients underwent complete ophthalmologic evaluation, assessment of best-corrected visual acuity with Early Treatment Diabetic Retinopathy Study (ETDRS) chart, reading speed with Minnesota Low-Vision Reading Test (MN Read) [7], SD-OCT and autofluorescence in SPECTRALIS Heidelberg Engineering equipment. Maia (Macular Integrity Assessment) microperimeter (CentreVue, Padova, Italy) equipment was used to determine the fixation area used by the patient and the retinal sensitivity. The Maia has a high-resolution fundus camera of 1024 × 1024 pixels and a high-frequency eye tracking system. This provides light stimuli in an accurate and repeatable way on precise areas of the retina, evaluating retinal sensitivity in specific points in a reliable and reproducible way. It also offers a map of the fixation area used by the patient (Fig. 2).

**Fig. 3:**
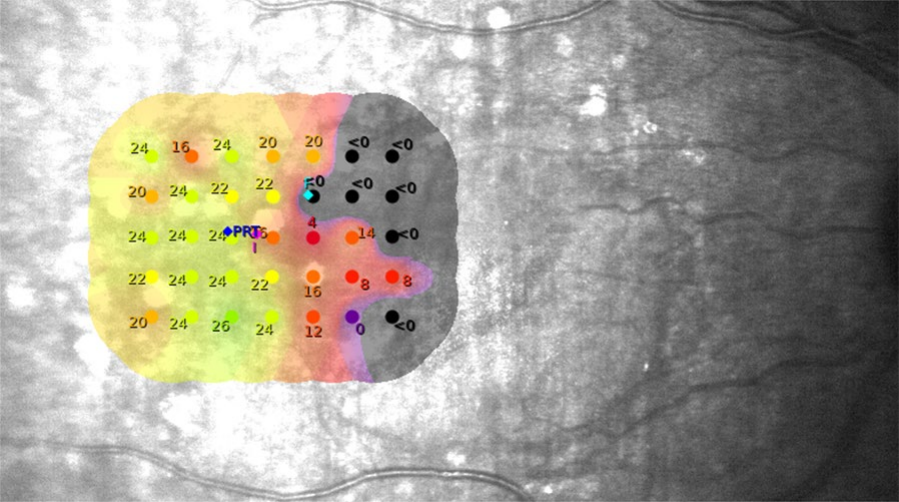
**Sensitivity map**. Based on this map, the selection of PRT (*dark blue dot*) is done. The patient is trained in order to direct its fixation to the *dark blue dot*. The *light blue dot* is the PRL, which corresponds to the average of fixation points used by the patient

Visual rehabilitation was developed so that the patient can establish the best possible fixation in the best area of retinal sensitivity. Considering the results of microperimetry and diagnostic images, the researchers selected this new desired fixation point called Preferred Retinal Target (PRT) (Fig. 3). The PRT selection has three fundamental premises that serve in the case that there are several points of the retina with good sensitivity, first we chose the point closest to the fovea and closest to the PRL, taking into account secondly that there would be an area beyond an isolated point with good sensitivity, and thirdly that this area would be arranged horizontally, which facilitates visual tasks, especially reading. Also the OCT was useful to check the integrity of the ellipsoid line in the areas of better sensitivity, however not being the objective of this investigation was not deepened in it. In some cases (7 of 18 patients), PRT could match the fixation point already set by the patient (PRL), in which case training was directed to enhance its stability.

**Fig. 4:**
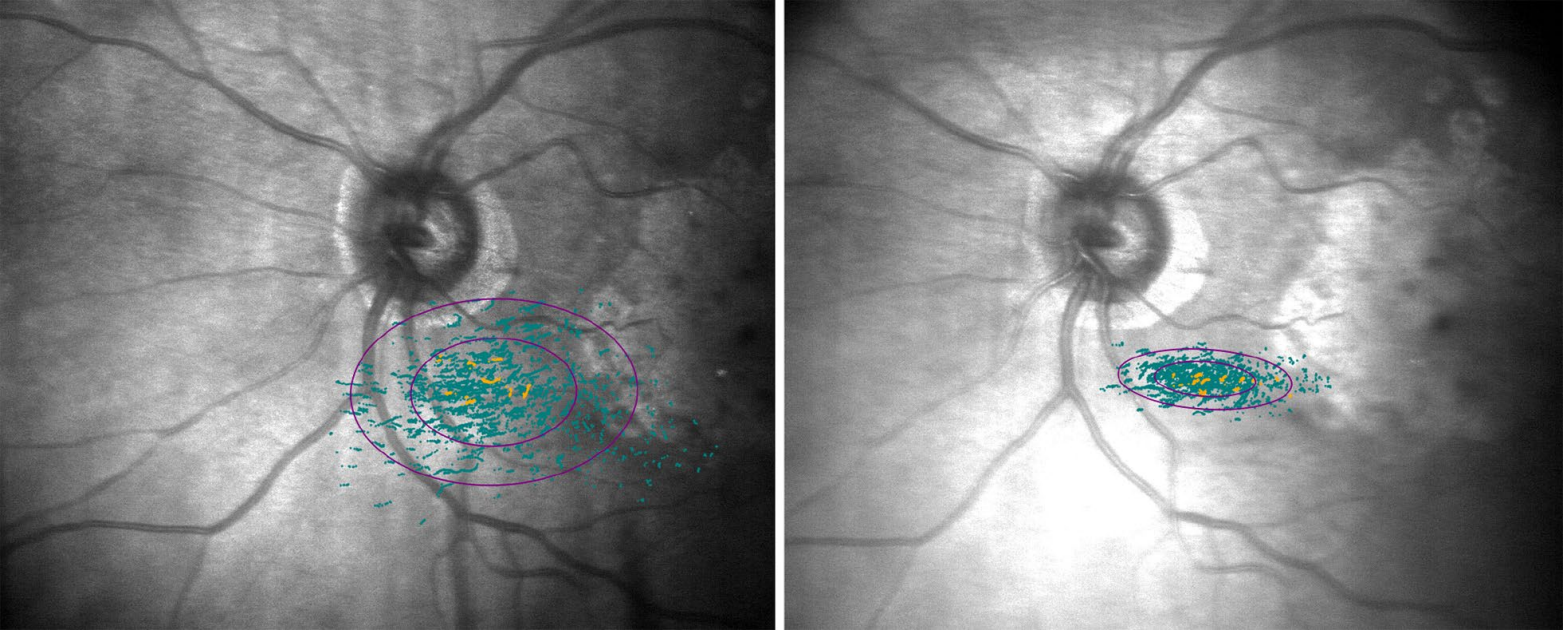
Change of fixation area used by the patient after training sessions

To achieve this purpose, the visual rehabilitation program includes 10 min training sessions in the better eye, twice per week for 8 weeks, this was based on the visual training experience of similar studies [6] (Fig. 4). Concluding this period, one week later, visual acuity tests, reading speed, and microperimetry were repeated to compare results before and after visual training therapy.

**Fig. 5:**
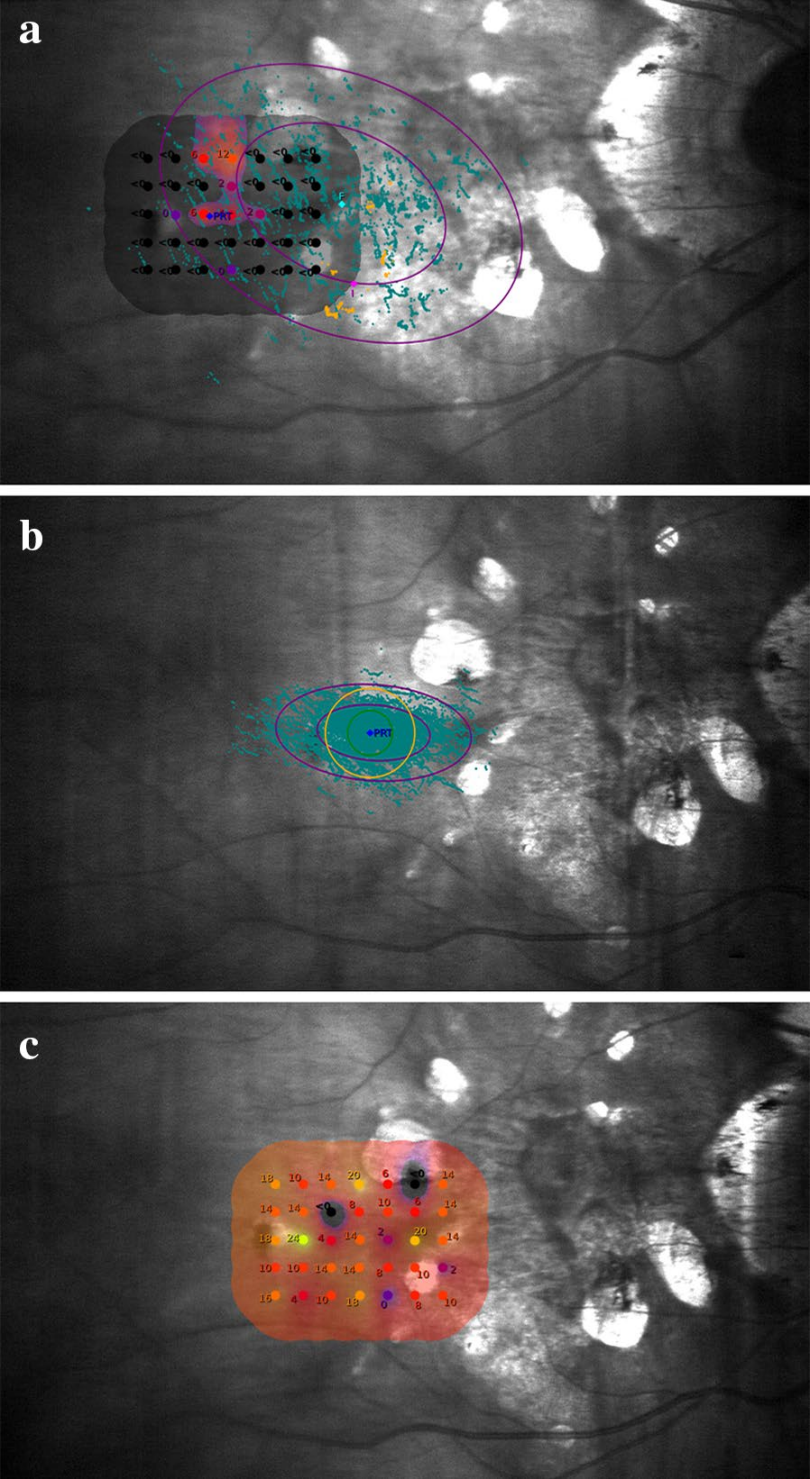
**a** Sensitivity map before training where it can be observed the difficulty in the fixation, PRT is selected in the area where sensitivity is observed. *Large ellipse* corresponds to 95% BCEA. **b** Trained fixation during training sessions. **c** New sensitivity map after rehabilitation, the same evaluated area as (**a**)

Visual acuity with ETDRS chart was converted to LogMAR scale for analysis. Reading speed, using both eyes, calculated in words per minute with the Mn Read Test, was compared with the same font size used before and after training. The “follow-up” option of Maia was used to document changes in retinal sensitivity; thus, the same points were evaluated before and after therapy. At each point of the retina, the sensitivity threshold was determined, and the average sensitivity threshold was taken into account for analysis (Fig. 5).

The fixation stability was quantified by the P1 and 95% Bivariate Contour Ellipse Area (BCEA) values. P1 expresses in percentage the number of fixation points that are within the area of a circle with 1-degree diameter, a P1 value greater than 75% indicates a stable fixation. 95% BCEA value establishes the ellipse area, expressed in square degrees, comprising 95% of the fixations points used by the patient during the test (Fig. 5). Therefore, higher P1 values and lower 95% BCEA values indicate a better fixation capacity.

### Data analysis and interpretation

For the statistical analysis, data were included in GraphPad Prism software 6.0 version. All variables were quantitative variables. D’Agostino and Pearson Normality test was done, so thereafter a Wilcoxon signed rank test was performed for he analysis of P1 values and reading speed; parametric paired “t” test was performed for the analysis of visual acuity, retinal sensitivity and % 95 BCEA values. *p* values ≤0.05 were considered statistically significant.

## Results

A total of 18 patients were included, 15 (83%) women with average age of 77 ± 8.2 years old (range between 65 and 92 years old) (Table 1).

**Table 1:** Basal characteristics

Patient	Sex	Age	V.A. eye to treat	V.A. other eye
1	F	70	0.4	0.4
2	F	77	0.4	2.1
3	F	85	0.4	1
4	F	77	1.1	1
5	M	74	1	1.4
6	M	68	0.7	1.3
7	F	69	0.6	1
8	F	65	0	1.9
9	F	73	1	1
10	F	75	0.6	1.3
11	M	92	1.1	1.4
12	F	82	0.5	0.8
13	F	91	1.1	2.1
14	F	67	0.2	0.6
15	F	73	1.5	1.6
16	F	75	0.5	0.7
17	F	89	0.6	1.4
18	F	82	0.3	1.3

*V.A.* visual acuity in LogMAR scale, *M* male, *F* female

The initial visual acuity of the eye to treat was on average 0.7 ± 0.4 LogMAR (0–1.5 LogMAR); one week after training, visual acuity was on average 0.6 ± 0.4 LogMAR (0–1.3 LogMAR) (*p* = 0.006) (Table 2).

**Table 2:** Visual acuity and reading speed

Patient	Initial V.A.	Final V.A.	Initial reading speed*	Final reading speed*
1	0.4	0.4	38	68
2	0.4	0.3	98	110
3	0.4	0.2	64	54
4	1.1	1.1	57	48
5	1	1.1	65	41
6	0.7	0.5	80	228
7	0.6	0.5	NA	NA
8	0	0	NA	NA
9	1	0.8	41	72
10	0.6	0.5	0	46
11	1.1	1	37	103
12	0.5	0.5	42	61
13	1.1	1.1	40	68
14	0.2	0.1	NA	NA
15	1.5	1.3	0	0
16	0.5	0.5	NA	NA
17	0.6	0.6	51	36
18	0.3	0.3	53	31

*V.A* visual acuity in LogMAR scale, *NA* not available information

* Reading speed with both eyes (Mn read test) in words per minute

Mean initial and final visual acuity for the untreated eye was 1.2 ± 0.4 LogMAR.

The initial reading speed calculated for both eyes was 47 ± 26 wpm (0–98 wpm), and the final reading speed was 69 ± 54 wpm (0–228 wpm) (*p* = 0.04).

Retinal sensitivity before and after training did not change significantly, with initial values of 14.1 ± 4.9 dB (6.1–22.3 dB) and the final values of 14.6 ± 4.3 dB (8.5–21.8 dB) (*p* = 0.4).

There was a improvement in fixation stability with initial values of P1 of 45 ± 32% (6–92%) and final values of P1 of 51 ± 29% (13–97%) *p* = 0.05. Fixation area also decreased significantly with baseline values of 95% BCEA = 43 ± 44 Square grades (3–130 Square grades) versus final values of 25 ± 21 Square grades (1–72 Square grades) *p* = 0.02 (Tables 3, 4).

**Table 3:** Fixation capacity and retinal sensitivity

Patient	Initial P1	Final P1	Initial 95% BCEA	Final 95% BCEA	Initial threshold*	Final threshold*
1	60	50	10.7	12.9	22.3	19.6
2	92	92	3.0	2.8	6.9	8.5
3	92	97	3.5	1.4	15.9	15.6
4	54	55	14.1	12.5	20.1	21.3
5	14	27	71.1	29.1	15.6	11.6
6	72	93	8.3	3.1	19.2	21.8
7	6	17	130.2	72.1	18.3	17.6
8	87	73	7.5	11.5	12.3	12.5
9	34	36	25.3	30.5	14.9	12.9
10	36	66	25.8	7.9	20.6	21
11	11	37	92.1	17.7	9.2	13.2
12	9	22	117.8	50.4	10.9	11.9
13	16	14	53.3	52.3	13.1	14.2
14	84	87	5.2	3.8	17.9	18.8
15	17	13	56.6	59.6	6.9	9.7
16	81	78	7.7	10.2	7.9	9.9
17	10	33	122.7	37.3	8.9	10.9
18	36	30	22.7	29.8	14.1	11.8

P1: expresses in percentage the number of fixation points that are within the area of a circle with 1-degree diameter. 95% BCEA (Bivariate Contour Ellipse Area) value establishes the ellipse area, expressed in square degrees, comprising 95% of the fixations points used by the patient during the test

* Average retinal sensitivity threshold in decibels

**Table 4:** Mean values and statistical significance

	Mean	SD (±)	*p* value
Initial visual acuity	0.7 LogMAR	0.4	0.006
Final visual acuity	0.6 LogMAR	0.4	
initial reading speed	47 wpm	26	0.04
Final reading speed	69 wpm	54	
Initial retinal sensitivity	14.1 dB	4.9	0.4
Final retinal sensitivity	14.6 dB	4.3	
Initial P1	45%	32	0.05
Final P1	51%	29	
Initial 95% BCEA	43°	44	0.02
Final 95% BCEA	25°	21	

All data included refers to the treated eye, except for the reading speed, which was with both eyes

*wpm* words per minute, *dB* decibels, *SD* standard deviation

## Discussion

The results presented demonstrate that the extrafoveal fixation capacity can be improved by training; moreover, in our patients, there was also a significant improvement in visual acuity and reading speed. It is known that in order to detect the details of an object, fixation is required, so it occurs physiologically on the fovea, it´s also described in the literature that the improvement in fixation implies an improvement in the visual capacity [8].

The final visual tests were performed at an early stage after training, so we can attribute the changes in these tests directly to the therapy, in addition to avoiding the bias that could cause the natural evolution of the disease.

Training effectiveness was explored using a new microperimeter, although there are studies that show that visual acuity can be improved using it in patients with successful closure of macular hole [9], to date, there are no publications in existence demonstrating the effectiveness of this device in patients with AMD. However, the results of our work are comparable with studies of visual rehabilitation with MP-1 microperimeter, where patients with AMD, myopic macular degeneration and other macular diseases showed improvements in visual acuity and fixation stability [5, 10, 11].

In our study, although Fixation stability improvement is questionable with *p* = 0.05 for P1 values changes, parameters that measure fixation capacity improved in our patients, and improved significantly with *p* = 0.02 for 95% BCEA.

There was proof that reading speed increased, after visual training, in a sample of six patients with macular degeneration [5], we also found an increase in reading speed. The reading speed tests were performed for each eye separately and in binocular form, for their analysis and interpretation we prefer to take into account the speed of binocular reading since in this way it is closer to a real scenario, since patients usually do not occlude an eye when reading.

Vingolo et al. found improvements in retinal sensitivity in a sample of five patients with different macular diseases after training with microperimetry [8], in our work, there were no significant changes in retinal sensitivity after training; this suggests that, despite being a subjective test, it is a reliable and reproducible diagnostic element. Based on this, we can understand the utility of microperimetry in other areas besides visual rehabilitation and why a variety of research studies focus on new treatments for macular diseases and the effectiveness of microperimetry [12–14]. Additionally, it has been proved that microperimetry is a useful tool for the follow-up of patients with diabetic retinopathy [15–18], central serous chorioretinopathy [19, 20], uveitic macular edema [21], macular dystrophies such as Stargardt disease [22], and AMD, in the latter evidencing a decrease in retinal sensitivity and fixation quality when the disease progresses [23].

Unlike what is reported in other studies [6, 8], in wich bilateral training was done we performed monocular training to avoid adverse effects such as diplopia, considering that not in all cases, were areas of retinal correspondence with good sensitivity found.

Visual training via microperimetry is controlled by the rehabilitator; guided by a sound system, the patient is encouraged to find and maintain the fixation on an established point (PRT). This requires that the patient have good comprehension skills and understanding of the tests and the process; furthermore, requires time availability and motivation, important factors in view of the advanced age of most patients who are candidates for this type of training.

The training process is static and its functionality should also be assessed during dynamic situations, as they occur in everyday life, whether on moving objects or tasks involving eye movements such as reading. The duration of training has not been established to obtain optimal results; neither when a new training, if required, should be done.

We think, from the experience of some patients, that visual training improves ability in daily tasks such as face recognition and walking, unfortunately we do not measure quality of life, but it will be important to do so in future studies.

It is interesting to note that not all cases showed improvement in the variables studied; therefore, more research is needed to determine which characteristics predict a favorable outcome in the patient with this kind of treatment.

## Conclusions

Visual training via microperimetry in patients with age-related macular degeneration is effective in improving fixation stability, reading speed, and visual acuity measured one week after training is completed, which is very important for these patients who suffer from a progressive disabling condition without effective treatment to date.
